# Prediction of Survival and Analysis of Prognostic Factors for Patients With Combined Hepatocellular Carcinoma and Cholangiocarcinoma: A Population-Based Study

**DOI:** 10.3389/fonc.2021.686972

**Published:** 2021-07-16

**Authors:** Jitao Wang, Zhi Li, Yong Liao, Jinlong Li, Hui Dong, Hao Peng, Wenjing Xu, Zhe Fan, Fengxiao Gao, Chengyu Liu, Dengxiang Liu, Yewei Zhang

**Affiliations:** ^1^ School of Medicine, Southeast University, Nanjing, China; ^2^ Xingtai Institute of Cancer Control, Xingtai People’s Hospital, Xingtai, China; ^3^ Department of Infection Management, Xingtai General Hospital of North China Healthcare Group, Xingtai, China; ^4^ Department of Hepatobiliary and Pancreatic Surgery, The Second Affiliated Hospital of Nanjing Medical University, Nanjing, China

**Keywords:** combined hepatocellular carcinoma and cholangiocarcinoma, overall survival, nomogram, prognostic factors, population-based study, Surveillance Epidemiology and End Results database

## Abstract

**Background:**

Combined hepatocellular carcinoma and cholangiocarcinoma (CHC) is an uncommon subtype of primary liver cancer. Because of limited epidemiological data, prognostic risk factors and therapeutic strategies for patients with CHC tend to be individualized. This study aimed to identify independent prognostic factors and develop a nomogram-based model for predicting the overall survival (OS) of patients with CHC.

**Methods:**

We recruited eligible individuals from the Surveillance, Epidemiology, and End Results (SEER) database between 2004 and 2015 and randomly divided them into the training or verification cohort. Univariate and multivariate analyses were performed to identify independent variables associated with OS. Based on multivariate analysis, the nomogram was established, and its prediction performance was evaluated using the consistency index (C-index) and calibration curve.

**Results:**

In total, 271 patients with CHC were included in our study. The median OS was 14 months, and the 1-, 3-, and 5-year OS rates were 52.3%, 27.1%, and 23.3%, respectively. In the training cohort, multivariate analysis showed that the pathological grade (hazard ratio [HR], 1.26; 95% confidence interval [CI]: 0.96–1.66), TNM stage (HR, 1.21; 95% CI: 1.02 - 1.44), and surgery (HR, 0.26; 95% CI: 0.17 - 0.40) were independent indicators of OS. The nomogram-based model related C-indexes were 0.76 (95% CI: 0.72 - 0.81) and 0.72 (95% CI: 0.66 - 0.79) in the training and validation cohorts, respectively. The calibration of the nomogram showed good consistency of 1-, 3-, and 5-year OS rates between the actual observed survival and predicted survival in both cohorts. The TNM stage (HR, 1.23; 95% CI: 1.01 - 1.49), and M stage (HR, 1.87; 95% CI: 1.14 3.05) were risk factors in the surgical treatment group. Surgical resection and liver transplantation could significantly prolong the survival, with no statistical difference observed.

**Conclusions:**

The pathological grade, TNM stage, and surgery were independent prognostic factors for patients with CHC. We developed a nomogram model, in the form of a static nomogram or an online calculator, for predicting the OS of patients with CHC, with a good predictive performance.

## Introduction

Combined hepatocellular carcinoma and cholangiocarcinoma (CHC) is a rare tumor subtype, it accounts for only 0.4%–14.2% of primary liver malignancies, and it has characteristics of hepatocellular carcinoma (HCC) and cholangiocarcinoma (CC) (1–3). In a large population-based study, the overall incidence of CHC was 0.05 per 100,000 person-years between 2004 and 2014, and its incidence and mortality have increased in recent years (1). The number of patients diagnosed with CHC almost doubled during 2004–2007 and 2012–2015, and patients with CHC more often had advanced T3–T4 stage cancer (57.0%) based on the guidelines of the American Joint Committee on Cancer (AJCC) and had a grim prognosis (2–5). The prognosis of CHC was reported as comparable to that of ICC but was worse than that of HCC (6–10), and patients with CHC have a lower survival rate than those with both the aforementioned malignancies (11–14). Therefore, the survival and prognosis of patients with CHC remain significant concerns.

Despite progress in treatment strategies, CHC is still considered an aggressive liver cancer with a poor prognosis and negligible improvement in recent years (15, 16). The main treatments for CHC include liver resection (LR) and liver transplantation (LT). Complete LR is considered to be the first-line treatment strategy for resectable CHC; however, the median overall survival (OS) of patients with CHC who have undergone surgery was only approximately 25–35.4 months (12, 13, 17–19). LT is another surgical option that may offer the only chance for long-term survival. Although LT has a survival advantage for patients with HCC, transplantation for CHC remains controversial (3, 16, 19).

The AJCC TNM staging system is widely used to assess the severity and predict the prognosis of patients with HCC or ICC (20). Although the TNM staging system has been confirmed to be a prognostic system for CHC (2, 21), its accuracy was not as remarkable as a serological model (22). However, many studies have shown that several independent risk factors, including age (23), race (5, 9), alpha-fetoprotein (AFP) status (23), cirrhosis (4), and treatment strategies (1, 5, 9, 24–26), affect the survival and prognosis of patients with CHC. At present, some single-center studies have constructed many nomogram prediction models for CHC (22, 27–30). Furthermore, studies have recently used the Surveillance, Epidemiology, and End Results (SEER) database to describe incidence trends and clinical outcomes of patients with CHC (1, 5); however, there was a lack of a nomogram to predict long-term survival.

Thus, this study aimed to analyze potential risk factors associated with the prognosis of patients with CHC and develop and validate a prognostic nomogram to enable clinicians to make better personalized decisions for treating patients with CHC.

## Methods

### Study Design and Patients

Our study collected clinical data of patients with CHC from the SEER database. The inclusion criteria of the study were patients diagnosed between 2004 and 2015, the primary tumor site was the liver, and the International Classification of Diseases for Oncology, third edition code was 8180/3: combined HCC and CC. Diagnostically confirmed cases included in our study were required to have positive histology findings. The exclusion criteria were unknown histological grade, unknown tumor size, unknown marital status at diagnosis, unknown surgical treatment, or lack of complete survival months.

### Data Collection and Definition of Variables

The following clinical information was collected for further analysis: baseline demographics, including ethnicity, age at diagnosis, sex, marital status, OS, and survival status; tumor features such as tumor size, pathological grade, TNM stage [AJCC 6^th^ edition], T stage, M stage, N stage, and treatment strategies, including surgery at the primary site, chemotherapy recode, and radiotherapy recode.

Sex was classified as male or female. Ethnicity was categorized into three race groups: Caucasian, African American, and others. Patients were classified into two groups: ≤60 years and >60 years according to the patient’s age at diagnosis. Marital status at diagnosis was categorized as married, single (never married), divorced/separated, or widowed. Tumor size was classified into two groups: ≤5 cm or >5 cm. Surgical types were classified as no surgery, LR, or LT. LR included local destruction, wedge resection (or segmental resection), lobectomy, and unclear surgical type. For radiotherapy and chemotherapy, patients were classified as with, without, or unknown.

### Statistical Analysis

We randomly divided all eligible patients with CHC into two groups: the training cohort (n=270) and the validation cohort (n=101). The nomogram-based model was constructed using the training cohort and verified using the verification cohort. We identified clinical characteristics with p-values ≤0.1 in the univariate analysis and further included them in the multivariate analysis. The nomogram model was constructed with independent prognostic factors based on the multivariate Cox regression analysis (p<0.05), and the efficacy was assessed using the concordance index (C-index). Calibration plots of the nomogram-based model for 1-, 3-, and 5-year OS in the training and validation cohorts were created by comparing nomogram-predicted OS with actual observed OS. In addition, according to the optimal cut-off value of the nomogram-based model score in the training cohort, all patients with CHC were divided into two groups: low or high risk. Clinically, surgical treatment strategies are related to the tumor grade, tumor stage, and patient’s clinical characteristics. The OS of patients with CHC was analyzed using the Kaplan-Meier method, and the log-rank test was used to compare the different groups. Clinical information was extracted using SEER*Stat software version 8.3.8 (www.seer.cancer.gov/seerstat). The data were analyzed using IBM SPSS Statistics 25.0 (IBM Corp., Armonk, NY, USA) and R software version 3.5.0 (The R Foundation, https://www.r-project.org/). The optimal cut-off value of the nomogram-based model score was calculated using X-Tile software version 3.6.1 (Yale University School of Medicine) (31).

Quantitative variables are expressed as median (interquartile range [IQR]) and were compared using the unpaired two-tailed Student’s t-test or the Kruskal-Wallis test as appropriate. Categorical data are expressed as numbers (percentage) and were compared using the χ^2^ test or the Fisher exact test as appropriate. P-values <0.05 were considered statistically significant.

## Results

### Patient Demographics

**Figure 1:**
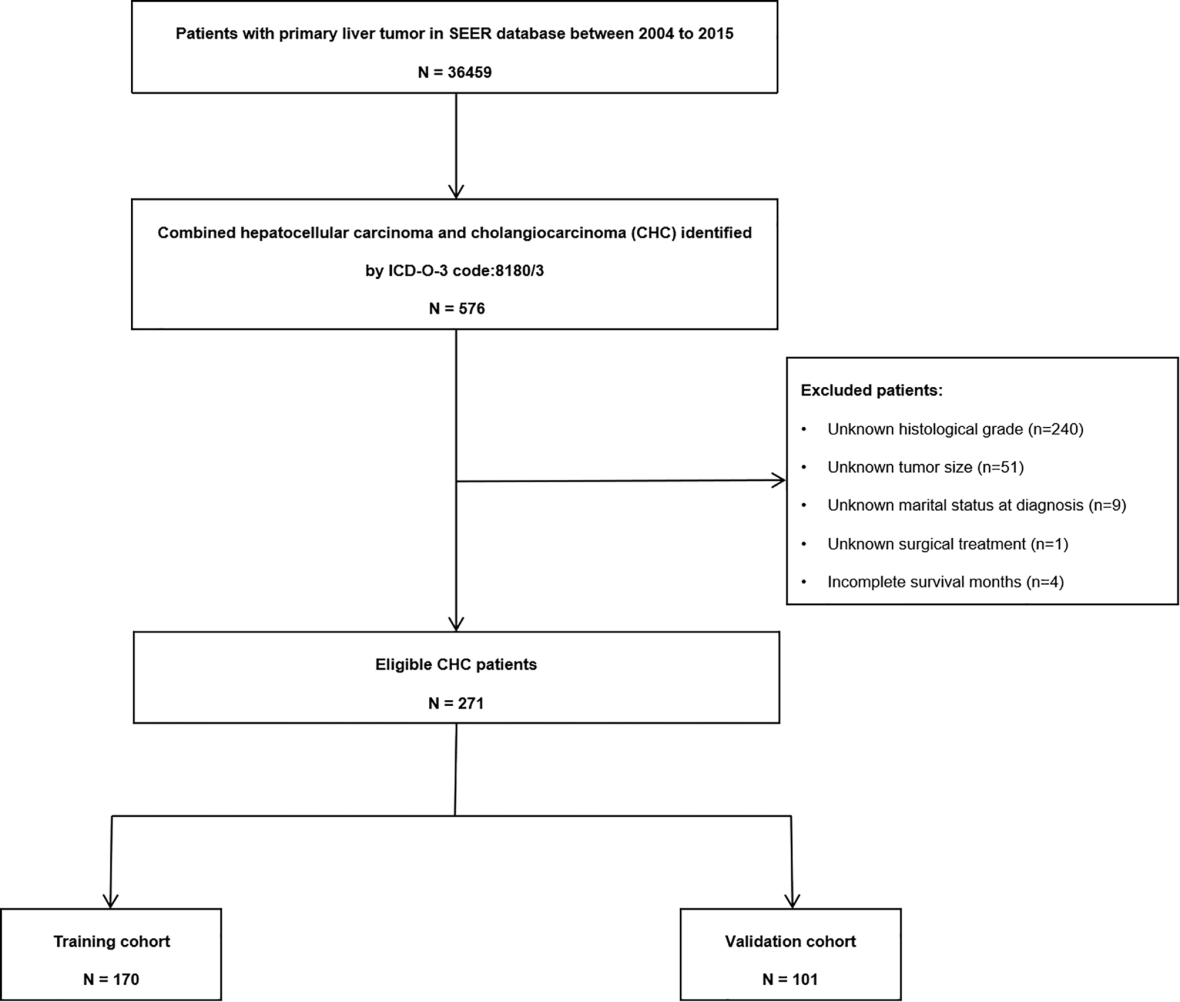
Flow chart of the combined hepatocellular carcinoma and cholangiocarcinoma patients.

According to the selection criteria, 271 patients (190 men; mean age, 61 years; age range, 14–88 years) were included in the final analysis (**Figure 1**). The most common race was Caucasian, accounting for 73.8% of the population. The median tumor size was 5.5 cm (IQR, 3.5–9.5 cm). Most patients presented with pathological grades III (57.2%) and II cancer (31.7%). A positive AFP status was found in 144 (53.1%) patients. Regarding treatment, most patients (161, 59.4%) underwent surgery, while 102 (37.6%) patients were administered chemotherapy, and 26 (9.6%) patients received radiotherapy. Baseline characteristics of the total, training, and validation cohorts are summarized in **Table 1**.

**Table 1:** Demographic and clinicopathologic characteristics, and overall survival of patients with combined hepatocellular carcinoma and cholangiocarcinoma.

Variables	Total cohort	Training cohort	Validation cohort
All patients	271	170	101
Year of diagnosis			
2004-2009	108 (39.8%)	73 (42.9%)	35 (34.5%)
2010-2015	163 (60.1%)	97 (57.0%)	66 (65.3%)
Gender			
Male	190 (70.1%)	114 (67.0%)	76 (75.2%)
Female	81 (29.8%)	56 (32.9%)	25 (24.7%)
Ethnicity			
Caucasian	200 (73.8%)	119 (70.0%)	81 (80.1%)
African–American	24 (8.9%)	14 (8.2%)	10 (9.9%)
Others	47 (17.3%)	37 (21.7%)	10 (9.9%)
Age at diagnosis			
≤60	129 (47.6%)	81 (47.6%)	48 (47.5%)
>60	142 (52.3%)	89 (52.3)	53 (52.4%)
Marital status			
Married	173 (63.8%)	108 (63.5%)	65 (64.3%)
Single	43 (15.9%)	25 (14.7%)	18 (17.8%)
Divorced/separated	33 (12.2%)	22 (12.9%)	11 (10.8%)
Widowed	22 (8.1%)	15 (8.8%)	7 (6.9%)
Tumor size			
≤ 5 cm	122 (45.0%)	72 (42.3%)	50 (49.5%)
> 5 cm	149 (54.9%)	98 (57.6%)	51 (50.4%)
Grade			
I	16 (5.9%)	12 (7.0%)	4 (3.9%)
II	86 (31.7%)	50 (29.4%)	36 (35.6%)
III	155 (57.1%)	98 (57.6%)	57 (56.4%)
IV	14 (5.1%)	10 (5.8%)	4 (3.9%)
TNM stage			
I	86 (31.7%)	52 (30.5%)	5 (4.9%)
II	67 (24.7%)	36 (21.1%)	34 (33.6%)
III	62 (22.8%)	41 (24.1%)	31 (30.6%)
IV	42 (15.4%)	32 (18.8%)	21 (20.7%)
Unknown stage	14 (5.1%)	9 (5.2%)	10 (9.9%)
T stage			
T1	101 (37.2%)	61 (35.8%)	40 (39.6%)
T2	83 (30.6%)	47 (27.6%)	36 (35.6%)
T3	61 (22.5%)	45 (26.4%)	16 (15.8%)
T4	22 (8.1%)	15 (8.8%)	7 (6.9%)
TX	4 (1.4%)	2 (1.1%)	2 (1.9%)
N stage			
N0	218 (80.4%)	133 (78.2%)	85 (84.1%)
N1	35 (12.9%)	23 (13.5%)	12 (11.8%)
NX	18 (6.6%)	14 (8.2%)	4 (3.9%)
M stage			
M0	215 (79.3%)	129 (75.8%)	86 (85.1%)
M1	42 (15.4%)	32 (18.8%)	10 (9.9%)
MX	14 (5.1%)	9 (5.2%)	5 (4.9%)
Surgery			
No surgery	111 (40.9%)	95 (55.8%)	35 (34.6%)
Yes	160 (59.0%)	75 (44.1%)	66 (65.3%)
Chemotherapy			
Yes	102 (37.6%)	72 (42.3%)	30 (29.7%)
No/unknown	169 (62.3%)	98 (57.6%)	71 (70.2%)
Radiation			
Yes	26 (9.5%)	17 (10.0%)	9 (8.9%)
No/unknown	245 (90.4%)	153 (90.0%)	92 (91.0%)

### Survival Analysis

In the total cohort, the median OS was 14.0 months (95% confidence interval [CI]: 10.4–17.6 months), and the 1-, 3-, and 5-year OS rates were 52.3%, 27.1%, and 23.3%, respectively. The mortality rate within 1 year was 47.7% in the total cohort. Detailed information is shown in **Table 1**. Pathological grade, TNM stage, tumor size, T stage, N stage, M stage, and surgery were identified as significant indicators of OS in the univariate analysis of the training cohort (**Table 2**). Independent predictors of OS indicated in the multivariable analysis were pathological grade (hazard ratio [HR]: 1.26; 95% CI: 0.96–1.66; P=0.01), TNM stage (HR: 1.21; 95% CI: 1.02–1.44; P=0.03), and surgery (HR, 0.26; 95% CI: 0.17–0.40, P<0.01) (**Table 2**).

**Table 2:** Univariate analysis and multivariate analysis of factors of overall survival (OS) of combined hepatocellular carcinoma and cholangiocarcinoma.

Variables	Category	Univariate analysis			Multivariate analysis		
		HR	95% CI	P value	HR	95% CI	P value
Year of diagnosis	2004-2009 ^*^/2010-2015	0.80	0.56-1.14	0.22			
Gender	Male*/Female	0.87	0.59-1.28	0.48			
Ethnicity	Caucasian*/African–American/Others	0.91	0.73-1.14	0.49			
Age at diagnosis	≤60*/>60	1.07	0.75-1.54	0.70			
Marital status	Married*/Single/(Divorced/separated)/Widowed	0.99	0.78-1.26	0.92			
Tumor size	≤ 5 cm*/>5 cm	1.48	1.02-2.14	0.04	1.02	0.68-1.51	0.94
Grade	I*/II/III/IV	1.87	1.25-2.79	<0.01	1.26	0.96-1.66	0.01
TNM stage	I*/II/III/IV/Unknown stage	2.01	1.42-2.86	<0.01	1.21	1.02-1.44	0.03
T stage	T1*/T2/T3/T4/TX	1.46	1.23-1.72	<0.01	1.16	0.95-1.43	0.12
N stage	N0*/N1/NX	1.66	1.27-2.17	<0.01	0.96	0.62-1.48	0.84
M stage	M0*/M1/MX	1.92	1.47-2.52	<0.01	1.25	0.78-2.03	0.36
Surgery	Yes*/No surgery	0.21	0.14-0.30	<0.01	0.26	0.17-0.40	<0.01
Chemotherapy	Yes*/(No/unknown)	1.13	0.78-1.62	0.52			
Radiation	Yes*/(No/unknown)	1.01	0.55-1.84	0.98			

HR, hazard ratio; CI, confidence interval; *reference category.

### Nomogram for Predicting OS

**Figure 2:**
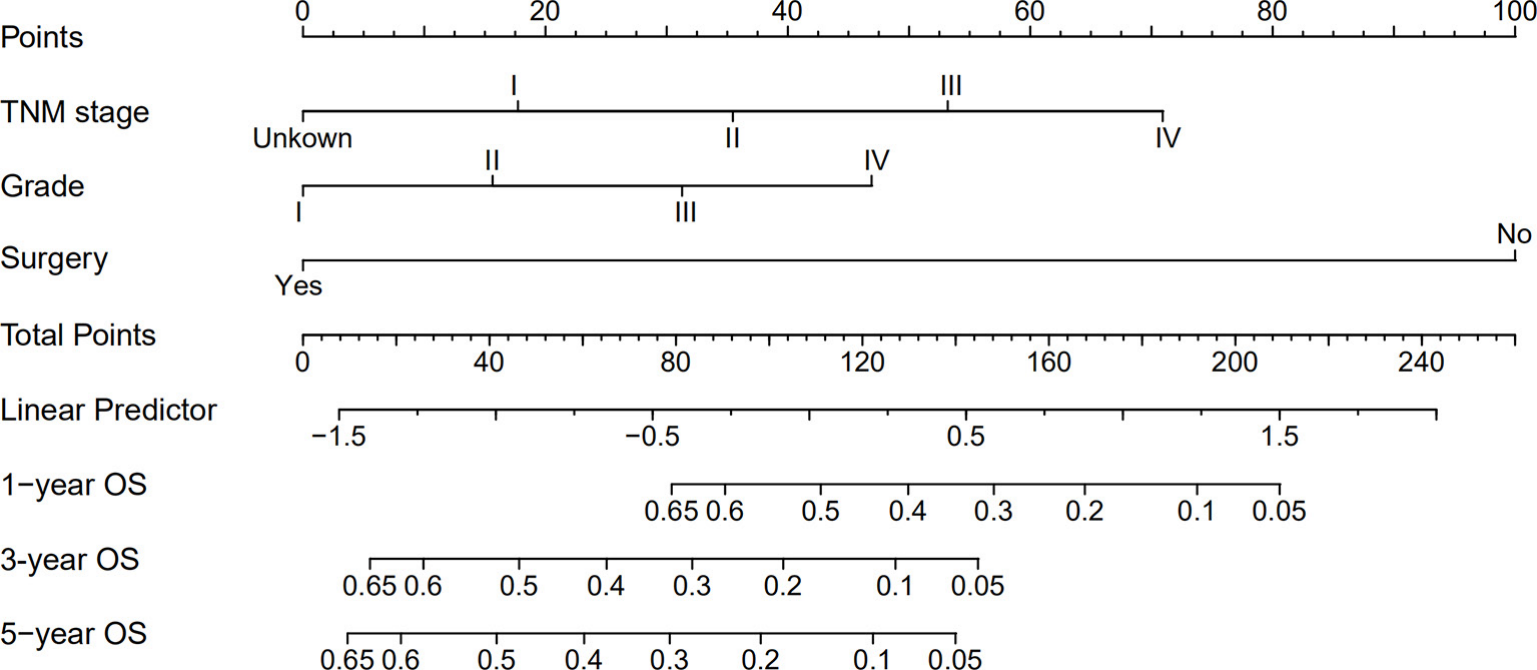
Nomogram for predicting 1-, 3- and 5-year overall survival (OS) of combined hepatocellular cholangiocarcinoma patients.

**Figure 3:**
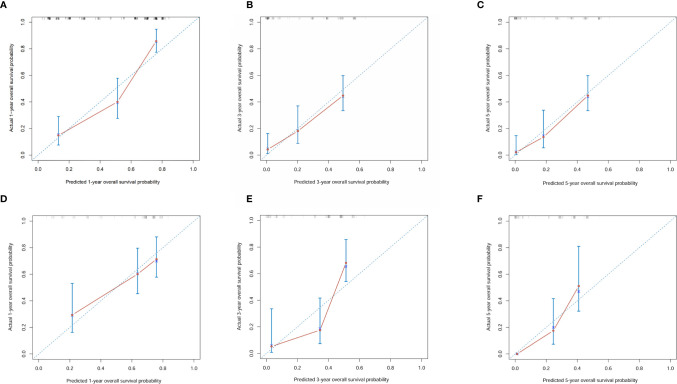
Calibration curves for 1-, 3- and 5-year overall survival of combined hepatocellular carcinoma and cholangiocarcinoma patients in the training cohort and validation cohort. **(A)** Calibration curves for 1-year overall survival of combined hepatocellular carcinoma and cholangiocarcinoma patients in the training cohort; **(B)** Calibration curves for 3-year overall survival of combined hepatocellular carcinoma and cholangiocarcinoma patients in the training cohort; **(C)** Calibration curves for 5-year overall survival of combined hepatocellular carcinoma and cholangiocarcinoma patients in the training cohort; **(D)** Calibration curves for 1-year overall survival of combined hepatocellular carcinoma and cholangiocarcinoma patients in the validation cohort; **(E)** Calibration curves for 3-year overall survival of combined hepatocellular carcinoma and cholangiocarcinoma patients in the validation cohort; **(F)** Calibration curves for 5-year overall survival of combined hepatocellular carcinoma and cholangiocarcinoma patients in the validation cohort.

A nomogram was established based on all independent prognostic variables identified in the multivariate analysis (**Figure 2**). Our nomogram was virtually displayed for predicting 1-, 3-, and 5-year OS in the training cohort and was validated in the validation cohort. The nomogram exhibited a satisfactory performance for predicting OS with C-indexes of 0.76 (95% CI: 0.72–0.81) and 0.72 (95% CI: 0.66–0.79) in the training and validation cohorts, respectively. The calibration curves for the probability of 1-, 3-, and 5-year OS manifested an optimal consistency between the actual observation and the nomogram-based model prediction in the training and validation cohorts (**Figure 3**).

**Figure 4:**
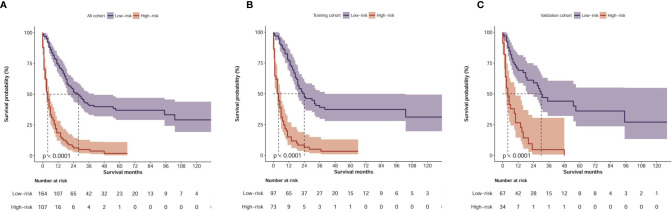
Kaplan–Meier curves of OS for risk classification based on the nomogram scores. **(A)** In all cohort; **(B)** In the training cohort; **(C)** In the validation cohort.

By applying the optimal cut-off value of the nomogram in the training cohort, we developed a risk stratification of OS. All patients with CHC were divided into the low-risk group (≤120 points) or high-risk group (>120 points) according to the nomogram-based model score. In the total cohort, Kaplan-Meier analysis showed that the median OS values were 28.0 months (95% CI: 20.5–35.5 months) and 4.0 months (2.7–5.7 months) in the low-risk and high-risk groups, respectively (P<0.001, **Figure 4A**). In the training cohort, the median OS values were 24.0 months (95% CI: 14.0–34.0 months) and 4.0 months (2.7–5.3 months) in the low-risk and high-risk groups, respectively (P<0.001, **Figure 4B**). In the validation cohort, the median OS values were 30.0 months (95% CI: 21.3–38.7 months) and 4.0 months (1.7–6.3 months) in the low-risk and high-risk groups, respectively (P<0.001, **Figure 4C**). An online calculator based on our nomogram model for clinicians and researchers to predict the survival probability of CHC patients by simply inputting clinical characteristics was developed (https://xingtai.shinyapps.io/CHC_DynNomapp/). Using the formula based on our nomogram model, the 5-year survival probability of the 10th patient in the verification cohort was calculated to be 34%, which is close to the result of the online calculator (36%, 95% CI: 0.23-0.59), which validated the accuracy of the calculator (**Figure S1**).

### Univariate and Multivariate Analyses of the Surgical Treatment Groups

**Figure 5:**
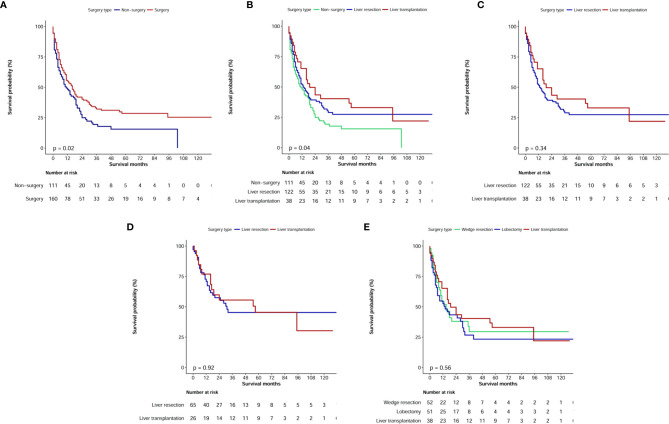
Kaplan–Meier overall survival curves of combined hepatocellular carcinoma and cholangiocarcinoma patients with different treatment strategies. **(A, B)** Survival analysis of different surgery type; **(C)** Comparison of prognosis of patients undergoing liver resection or liver transplant before and after propensity score matching; **(D)** Comparison of prognosis of patients undergoing liver resection or liver transplant in TNM I+II stage; **(E)** Survival analysis for specific surgical strategies.

The median OS values were 29 months (95% CI: 21.8–36.2 months) for patients with CHC who underwent surgical treatment (LR or LT) and 4 months (95% CI: 2.7–5.3 months) for patients with CHC who did not undergo surgical treatment (P<0.0001, **Figure 5A**). Therefore, when compared with no surgery, LR and LT significantly prolonged OS (**Figure 5B**). After excluding non-surgical patients, univariate analysis showed that the tumor size, pathological grade, TNM stage, T stage, N stage, M stage, AFP status, and chemotherapy were risk factors of prognosis (P<0.1). However, in the multivariate analysis, the TNM stage (HR, 1.22; 95% CI: 1.01–1.48) and M stage (HR, 1.83; 95% CI: 1.12–2.99) alone were independent predictors of OS in the surgical treatment group (**Table 3**).

**Table 3:** Univariate and multivariate analyses of prognostic factors of combined hepatocellular carcinoma and cholangiocarcinoma patients undergoing surgery.

Variables	Category	Univariate analysis			Multivariate analysis		
		HR	95% CI	P value	HR	95% CI	P value
Year of diagnosis	2004-2009 ^*^/2010-2015	1.02	0.69-1.51	0.93			
Gender	Male*/Female	1.11	0.73-1.69	0.64			
Ethnicity	Caucasian*/African–American/Others	1.00	0.78-1.30	0.99			
Age at diagnosis	≤60*/>60	1.09	0.85-1.40	0.50			
Marital status	Married*/Single/(Divorced/separated)/Widowed	1.17	0.89-1.55	0.27			
Tumor size	≤ 5 cm*/>5 cm	2.24	1.49-3.37	<0.01	1.52	0.96-2.40	0.07
Grade	I*/II/III/IV	1.53	1.14-2.06	<0.01	1.35	0.98-1.86	0.07
TNM stage	I*/II/III/IV/Unknown stage	1.46	1.21-1.78	<0.01	1.22	1.01-1.48	0.04
T stage	T1*/T2/T3/T4/TX	1.41	1.17-1.70	<0.01	1.02	0.82-1.28	0.86
N stage	N0*/N1/NX	2.29	1.67-3.16	<0.01	1.32	0.83-2.12	0.24
M stage	M0*/M1/MX	2.55	1.85-3.51	<0.01	1.83	1.12-2.99	0.02
Surgery type	Yes*/No surgery	0.81	0.51-1.27	0.35			
Chemotherapy	Yes*/(No/unknown)	1.59	1.07-2.36	0.02	0.95	0.60-1.48	0.81
Radiation	Yes*/(No/unknown)	1.65	0.88-3.10	0.12			

HR, hazard ratio; CI, confidence interval; *reference category.

### Surgical Treatment Strategies

In the surgical treatment cohort, 122 patients underwent surgical resection (including four cases of local tumor destruction and six cases of heat radiofrequency ablation) and 38 patients underwent LT. Further analysis showed that the median OS values were 13.0 months (95% CI: 7.9–18.1 months) in patients who underwent LR and 19.0 months (95% CI: 8.3–29.7 months) in patients who underwent LT; however, no significant difference was observed (P=0.34, **Figure 5C**).

Regarding clinical practice, surgeons have recommended that patients with TNM stage I+II cancer should undergo LR or LT. Therefore, in our cohort of patients with AJCC stage I+II cancer, we further analyzed the median OS of 26 patients who underwent LT, and it was estimated to be 57 months, which was longer than the median OS of 65 patients who received LR (31 months); this difference, however, was not significant (P=0.92, **Figure 5D**).

We further analyzed the difference in survival of patients with CHC who underwent different surgical strategies. Among 160 patients with CHC who underwent surgical treatment, 10 who received local destruction and nine who had an unclear surgical strategy were excluded from the final analysis. The median OS values for patients with CHC who underwent liver wedge resection, liver lobectomy, and LT were 15 months (8.3–21.7 months), 14 months (4.1–23.9 months), and 19 months (8.3–29.7 months), respectively. There was no significant difference among the three groups (P=0.56, **Figure 5E**). The pathological grade in the transplant group was significantly different compared with those in the lobectomy group (**Table 4**). There were no significant differences in age, sex, race, marital status, T stage, N stage, M stage, and TNM stage of patients between the lobectomy group or the wedge resection group and the LT group.

**Table 4:** The clinical characteristics of combined hepatocellular carcinoma and cholangiocarcinoma patients undergoing specific surgical strategies.

Variables	Liver transplant	Ref	Wedge resection	P value	Lobectomy	P value
All patients	38		52		51	
Year of diagnosis		Ref		0.01		0.26
2004-2009	21 (55.3%)		14 (26.9%)		22 (43.1%)	
2010-2015	17 (44.7%)		38 (73.1%)		29 (56.9%)	
Gender		Ref		0.16		0.20
Male	30 (78.9%)		34 (65.4%)		34 (66.7%)	
Female	8 (21.1%)		18 (34.6%)		17 (33.3%)	
Ethnicity		Ref		0.22		0.15
Caucasian	30 (78.9%)		36 (69.2%)		33 (64.7%)	
African–American	5 (13.2%)		5 (9.6%)		6 (11.8%)	
Others	3 (7.9%)		11 (21.2%)		12 (23.5%)	
Age at diagnosis		Ref		0.05		0.06
≤60	24 (63.2%)		22 (42.3%)		22 (43.1%)	
>60	14 (36.8%)		30 (57.7%)		29 (56.9%)	
Marital status		Ref		0.12		0.23
Married	23 (60.5%)		35 (67.3%)		30 (58.8%)	
Single	11 (28.9%)		7 (13.5%)		9 (17.6%)	
Divorced/separated	1 (2.6%)		7 (13.5%)		7 (13.7%)	
Widowed	3 (7.9%)		3 (5.8%)		5 (9.8%)	
Tumor size		Ref		0.13		0.07
≤ 5 cm	23 (60.5%)		23 (44.2%)		21 (41.2%)	
> 5 cm	15 (39.5%)		29 (55.8%)		30 (58.8%)	
Grade		Ref		0.19		0.01
I	3 (7.9%)		4 (7.7%)		0 (0.0%)	
II	17 (44.7%)		15 (28.8%)		15 (29.4%)	
III	18 (47.4%)		29 (55.8%)		29 (56.9%)	
IV	0 (0.0%)		4 (7.7%)		7 (13.7%)	
TNM stage		Ref		0.47		0.58
I	13 (34.2%)		17 (32.7%)		13 (25.5%)	
II	13 (34.2%)		10 (19.2%)		14 (27.5%)	
III	6 (15.8%)		13 (25.0%)		15 (29.4%)	
IV	4 (10.5%)		9 (17.3%)		7 (13.7%)	
Unknown stage	2 (5.3%)		3 (5.8%)		2 (3.9%)	
T stage		Ref		0.30		0.22
T1	15 (39.5%)		20 (38.5%)		15 (29.4%)	
T2	15 (39.5%)		12 (23.1%)		18 (35.3%)	
T3	5 (13.2%)		11 (21.2%)		16 (31.4%)	
T4	3 (7.9%)		7 (13.5%)		2 (3.9%)	
TX	0 (0.0%)		2 (3.8%)		0 (0.0%)	
N stage		Ref		0.69		0.20
N0	31 (81.6%)		39 (75.0%)		44 (86.3%)	
N1	6 (15.8%)		10 (19.2%)		3 (5.9%)	
NX	1 (2.6%)		3 (5.8%)		4 (7.8%)	
M stage		Ref		0.65		0.87
M0	32 (84.2%)		40 (76.9%)		42 (82.4%)	
M1	4 (10.5%)		9 (17.3%)		7 (13.7%)	
MX	2 (5.3%)		3 (5.8%)		2 (3.9%)	
Chemotherapy		Ref		0.07		0.08
Yes	9 (23.7%)		22 (42.3%)		21 (41.2%)	
No/unknown	29 (76.3%)		30 (57.7%)		30 (58.8%)	
Radiation		Ref		0.21		0.91
Yes	4 (10.5%)		2 (3.8%)		5 (9.8%)	
No/unknown	34 (89.5%)		50 (96.2%)		46 (90.2%)	

Ref, reference.

## Discussion

In this population-based study, we identified independent prognostic factors and constructed a prognostic nomogram-based model to predict the 1-, 3-, and 5-year OS of patients with CHC. The model facilitates accurate survival prediction, high-risk patient screening, and personalized treatment. An easy-to-use online calculation application with free access was provided (https://xingtai.shinyapps.io/CHC_DynNomapp/). A patient’s survival probability with 95% CI can be quickly obtained by entering three clinical characteristics.

Owing to the rarity of CHC, it is difficult to accurately assess the prognostic factors of CHC using data from a single institution. To date, few population-based studies have reported the clinical outcomes and prognostic risk factors for patients with CHC using the SEER database (1, 5, 32). However, in these studies, nearly half of the patients with CHC lacked data on the pathological grade, and there was no correlation between the pathological grade and survival of patients with CHC (1, 5), which could affect the accuracy and persuasiveness of the conclusions of the studies. More importantly, although prognostic risk factors have already been reported, previous studies did not provide a prognostic model to facilitate clinicians and patients to predict the prognosis of CHC accurately and individually (1, 5). Our study excluded patients with CHC who lacked or included uncertain important information (such as the pathological grade, tumor size, and presence of surgery) and therefore could more accurately reflect whether there are differences in survival between each group. To our knowledge, our study is the first to report that pathological grade is significantly correlated with the survival of patients with CHC, which is different from that reported in previous studies (1, 5).

In the past few decades, although the OS of patients with CHC has gradually improved, it remains to be at frustratingly poor. In our analysis, the 5-year OS rate was 23.3%, which was higher than that (10.5%) reported in a population-based study based on the SEER database conducted between 1988 and 2009 (9). This phenomenon has also been confirmed in our research. The OS of patients with CHC in 2010–2015 was better than that of patients with CHC in 2004–2009 (the 5-year survival rates were 28.3% and 19.8%, respectively); however, no significant difference was noted. The median survival in our cohort was 14 months, which was higher than that in two other large population-based studies (1, 5) (8 and 9 months); this was mainly attributed to a higher proportion of patients who underwent surgery in our cohort.

In the present study, the pathological grade, TNM stage, and surgical type were identified as independent prognostic factors, among which surgery was a particularly important factor affecting OS (2, 16, 25). The 5-year OS in patients with CHC who underwent surgery reached 28.5%, while it was only 15.6% in those who received non-surgical treatment. The pathological grade is considered to be an important prognostic indicator for many cancers, including CHC (33). The TNM staging system has been one of the most commonly used tumor staging systems and is proven to be suitable for patients with CHC (2). However, a recent study (22) showed that its predictive power may not be as good as other standards. Based on the multivariate analysis, our nomogram-based model included three important variables (pathological grade, TNM stage, and surgical type) and could accurately categorize patients with CHC into different prognostic groups.

Surgery has been the most important treatment that affects the survival of patients with CHC (1, 5, 24). To better analyze such patients, we further analyzed prognostic factors in the surgical cohort. Unlike the overall cohort, AFP status is an independent prognostic factor for patients with CHC who undergo surgery. Wang et al. also confirmed that higher serum AFP levels combined with imaging features was an independent risk factor for postoperative microvascular invasion (MVI) in patients with CHC and that patients with CHC who had MVI could have higher risks of recurrence early after surgery (34). This may suggest that in patients with CHC who undergo surgery, the AFP level should be actively monitored and evaluated.

There are some controversies about surgical strategies for patients with CHC. In the current study, patients who underwent LR and LT had significantly prolonged OS compared with those who did not undergo surgery, and they had comparable OS between the two treatment strategies. Furthermore, there was no significant difference among wedge resection, lobectomy, and LT treatment. However, the number of patients undergoing LR has increased over time, and the number of patients with CHC undergoing LR increased by 1.9 times between 2004–2009 and 2010–2015. This increase was not observed in patients with CHC who underwent LT. Between the periods of 2004–2007 and 2012–2015, the number of patients undergoing LT remained relatively stable. Groeschl et al. (32) also confirmed that although LT was another alternative treatment that resulted in better survival benefits for patients with CHC, the treatment effect was inferior to LT; this result may be related to the characteristics of CC. However, a recent multicenter retrospective study confirmed that regardless of the tumor burden, the clinical prognosis of LT was superior to that of LR in patients with CHC (24). Specifically, patients with CHC who underwent LT based on the Milan criteria had a better 5-year OS than those who underwent resection, but this was not a significant difference (70.1% and 49.7%, respectively; P=0.078). However, there was no significant difference in OS among CHC patients with tumor burden beyond University of California San Francisco (UCSF) criteria or within UCSF criteria but beyond Milan criteria. In our cohort of patients with TNM stage I+II cancer, the median OS of patients undergoing LT was longer than that of those undergoing LR (51 months and 31 months, respectively); however, there was no significant difference (P=0.92). This finding was more likely because of the statistical bias caused by the number of patients with CHC. Lunsford et al. confirmed that patients with CHC with low-grade, well-moderately differentiated tumors had excellent survival with a low risk for post-LT recurrence and seemed to benefit from LT (33). Therefore, doctors should remember to determine the tumor stage and pathological grade of patients with CHC before deciding surgical treatment strategies.

In this study, we constructed a nomogram-based model according to the multivariate analysis, which could categorize all patients with CHC into low-risk or high-risk prognostic subgroups. Our nomogram-based model performed well in predicting prognosis, and the C-index and calibration curves supported the survival prediction both in the training and validation groups. However, this study has some limitations. First, some important variables such as the AFP status, liver fibrosis score, health status, and underlying diseases had an excessive proportion of incomplete clinical information or were unavailable in the SEER database. Because there was no distinction between unacceptable and unknown chemotherapy/radiotherapy in the SEER database, we could not accurately analyze the effect of those variables on the survival of patients with CHC. Second, although our cohort was recruited from the SEER database, which is a high-quality, population-based cancer registry, our sample size was still relatively small owing to the rarity of CHC. Finally, although our nomogram showed good discrimination ability and a consistent calibration curve in both the training and internal verification cohorts, an external verification cohort for the nomogram-based model is still required.

## Conclusions

CHC has an extremely poor prognosis, and its prognosis has not improved in recent years. Our study demonstrated that pathological grade, TNM stage, and surgery type were independent prognostic factors for patients with CHC. LR and LT significantly prolonged OS compared with non-surgical treatment. Our nomogram showed good predictive performance, and therefore, it could be used to predict the prognosis of patients with CHC, along with screening for high-risk patients. Prediction models based on static nomograms or online prediction tools (available at https://xingtai.shinyapps.io/CHC_DynNomapp/) could accurately predict the survival probability of CHC patients.

## Data Availability Statement

Publicly available datasets were analyzed in this study. This data can be found here: Publicly available datasets were analyzed in this study. This data can be found here: https://seer.cancer.gov/data/.

## Ethics Statement

Ethical review and approval was not required for the study on human participants in accordance with the local legislation and institutional requirements. Written informed consent for participation was not required for this study in accordance with the national legislation and the institutional requirements.

## Author Contributions

JW and YZ designed the study. JW and ZL provided the databases. JW, ZL, YL, JL, HD, HP, WX, ZF, FG, CL, DL, and YZ assembled and analyzed the data. JW and ZL wrote the manuscript. All authors have contributed to the article and have approved the submitted version.

## Funding

The present study was funded by the National Natural Science Foundation of China (grant no. 81872255), the Key medical talents fund of Jiangsu Province (grant no.2016KJQWZ DRC-03), and the Southeast University–China Pharmaceutical University Cooperative Research Project (grant no. 2242019K3DZ06), the Hebei Provincial Key R&D Program Project (grant no.18277717D), the Hebei Provincial Health Commission Scientific Research Fund Project (grant no.20181612), and the Xingtai City Key R&D Program Project (grant no.2020ZZ026).

## Conflict of Interest

Author ZL was employed by the company, Xingtai General Hospital of North China Healthcare Group.

The remaining authors declare that the research was conducted in the absence of any commercial or financial relationships that could be construed as a potential conflict of interest.
