# Supplementary figures and images for: Prediction of Survival and Analysis of Prognostic Factors for Patients With Combined Hepatocellular Carcinoma and Cholangiocarcinoma: A Population-Based Study
Source: Front Oncol. 2021 Jul 16;11:686972. doi: 10.3389/fonc.2021.686972 (PMC8322675; doi:10.3389/fonc.2021.686972)

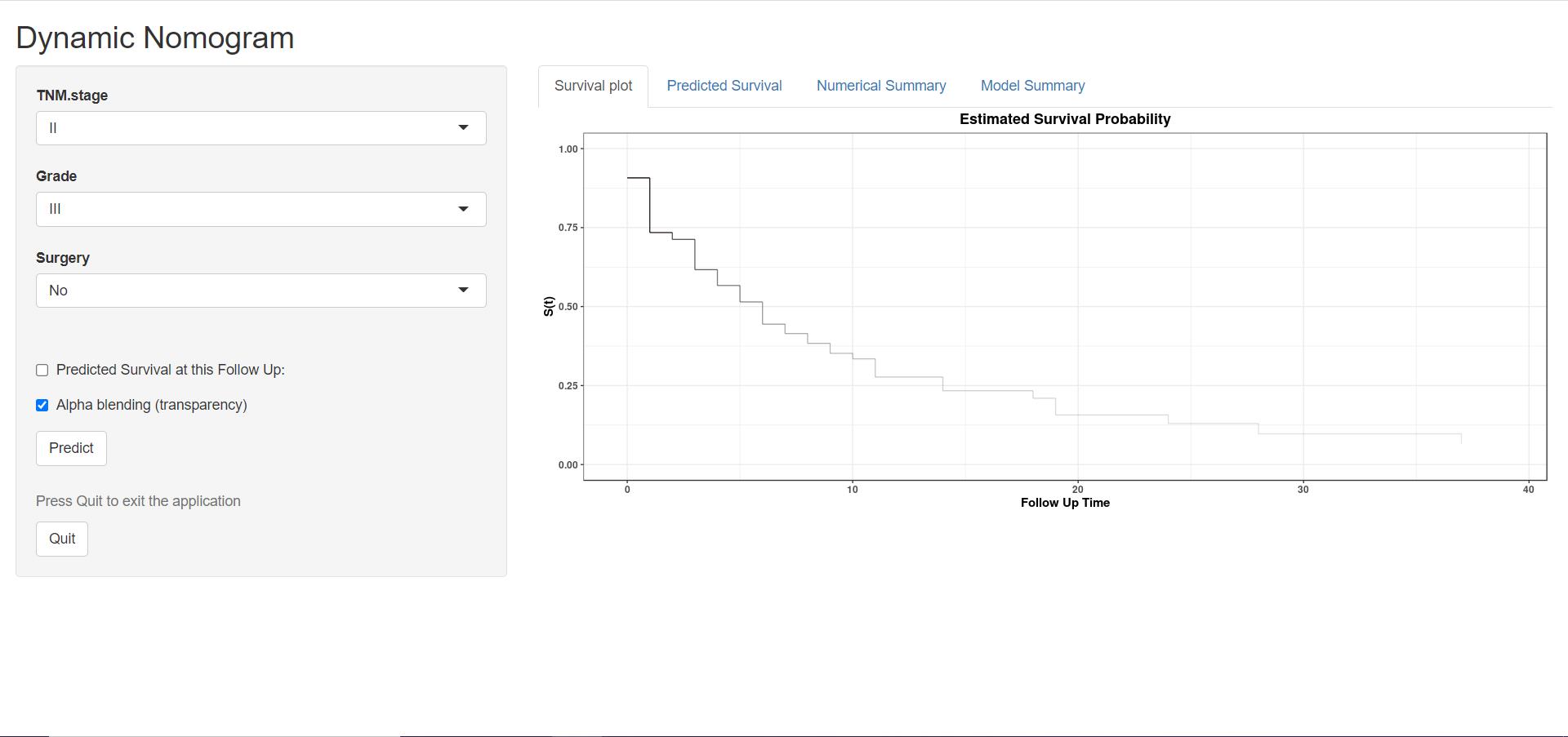

Supplement: Supplementary file 1 [file Image_1.jpeg]
